# Research status of gas sensing performance of Ti_3_C_2_Tx-based gas sensors: A mini review

**DOI:** 10.3389/fchem.2022.1037732

**Published:** 2022-10-03

**Authors:** Bo Peng, Xinlu Huang

**Affiliations:** College of Materials Science and Engineering, Chongqing Jiaotong University, Chongqing, China

**Keywords:** Ti_3_C_2_Tx, volatile organic compounds (VOCs) gases, composite materials, surface functionalization, sensing performance

## Abstract

Developing efficient gas sensing materials capable of sensitive, fast, stable, and selective detection is a requisite in the field of indoor gas environment monitoring. In recent years, metal carbides/nitrides (MXenes) have attracted attention in the field of gas sensing because of their high specific surface area, good electrical conductivity, and high hydrophilicity. Ti_3_C_2_Tx, the first synthesised MXene material, has also become the most popular MXene material owing to its low formation energy. In this paper, the latest progress in the application of Ti_3_C_2_Tx-based nanomaterials in the field of gas sensors is reviewed. Some challenges currently faced by Ti_3_C_2_Tx gas sensors are discussed, and possible solutions are proposed, focusing on the use of composite materials and surface functionalization methods to modify Ti_3_C_2_Tx nanomaterials to improve their sensing performance for the detection of gaseous volatile organic compounds. This study highlights the application prospects of Ti_3_C_2_Tx nanomaterials in gas sensors.

## Introduction

In recent years, with the acceleration of urbanization, the content of volatile organic compounds (VOCs) such as toluene (C_7_H_8_), formaldehyde (HCHO), ethanol (C_2_H_5_OH), and acetone (C_3_H_6_O) in the air has risen rapidly. Subsidence to form ground-level ozone endangers human health (Malakar et al., 2017; Maung et al., 2022; Mozaffar et al., 2020; Yue et al., 2021); the effects and exposure limits are presented in Table 1. Therefore, all sectors of society have focused on the use of gas sensors to monitor toxic and harmful gases in indoor and outdoor environments, where gas monitoring is widely adopted in industrial manufacturing and disease diagnosis (Chaudhary et al., 2022; Chen et al., 2020a; Wang et al., 2022a). Researchers have combined metal oxides (Hu et al., 2021; Peng et al., 2022), transition metal dichalcogenides (TMDs) (Sun et al., 2022; Xin et al., 2019), carbon-based materials (Liu et al., 2021), and some emerging two-dimensional (2D) materials for application in gas sensors to develop a series of sensitive and detection-selective gas sensors. However, although gas sensor materials such as metal oxides and conductive polymers possess good electrochemical performance and gas sensitivity, their working environment (200°C) is demanding, which exposes the defects of high power consumption and difficult application.

**TABLE 1:** Effects of various VOCs on humans.

Harmful gases	Major sources	Harm to human	Lowest exposure range for human
C_7_H_8_	cigarette, paint	Headache, vomiting, confusion	300 ppm
HCHO	volcanic gases, pesticides, paints, furniture	Blurred vision, vertigo	0.1 mg/m^3^
C_2_H_5_OH	industries	Paralysis of the nervous system, damage to the brain	3,300 ppm
C_3_H_6_O	petroleum refining, vehicle emissions	Difficulty breathing, corroded eyes	750 ppm for 15 min and 500 ppm for 8 h
CH_3_OH	Industrial workshop, Food processing plant	Affect the nervous system and blood system of human body	50 mg/m^3^
C_4_H_10_	Petroleum gas, natural gas and cracked gas	dizziness, headache, lethargy,coma	300 mg/m^3^
C₆H_15_N	Dyestuff, preservative, solvent	Cause pulmonary edema and even death	0.14 mg/m^3^

As a new material that was discovered only in 2011 (Naguib et al., 2011), MXene has a great potential in the sensor field owing to its unique morphology and good electrochemical properties (Zhang et al., 2018). Similar to graphene, MXene is a novel 2D-layered material composed of transition metal carbides/nitrides (Chaudhary et al., 2022). The transition metal carbide Ti_3_C_2_Tx, the first MXene material synthesised by etching from the MAX phase, has also become the most popular MXene material because of its relatively low formation energy (Naguib et al., 2011).

Ti_3_C_2_Tx has a higher specific surface area, and the contact surface with the air is larger under the same mass condition, which helps to improve the performance of the sensor (Li et al., 2021). Some experiments have demonstrated the feasibility of Ti_3_C_2_Tx in gas sensing (Koh et al., 2019; Lee et al., 2017). In this case, Ti_3_C_2_Tx is expected to prepare efficient and reliable gas sensors at room temperature. However, scholars have also found that traditional Ti_3_C_2_Tx materials possess a large number of -F, -OH or -O terminal groups, which make them degrade rapidly in a humid environment. This also exposes the problems of slow response, slow recovery, easy oxidation and poor stability of Ti_3_C_2_Tx sensors under wet conditions (Chae et al., 2019), which is also a huge challenge for Ti_3_C_2_Tx gas sensors at this stage.

Many review articles on Ti_3_C_2_Tx materials have been published, where the main focus has been the fields of biomedicine and photocatalysis. The application of Ti_3_C_2_Tx in gas sensors has not received much attention; in particular, the literature on the detection of VOCs gas remains very limited. In this review, the efficacy of different methods for improving the performance of sensors based on Ti_3_C_2_Tx materials is analysed, and the mechanisms are discussed. This study provides guidance for developing more efficient Ti_3_C_2_Tx-based sensors.

## Pristine Ti_3_C_2_Tx

**FIGURE 1:**
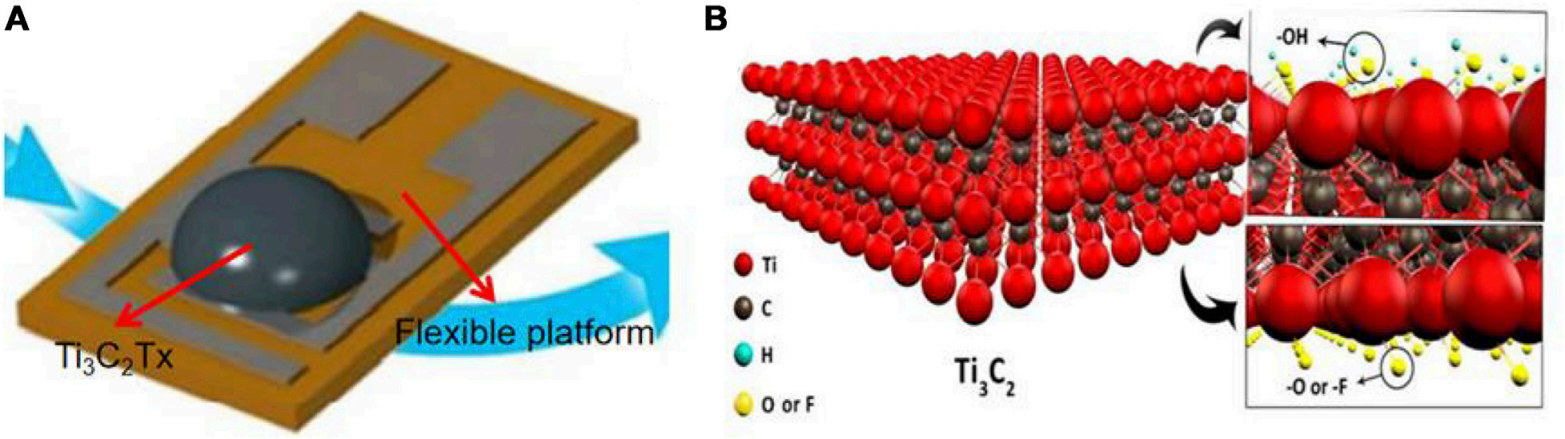
**(A)** Solid solution pouring method to prepare the sensor.**(B)** Ti3C2Tx structure and surface functional groups (adapted from Lee et al., 2017).

In 2017, Lee et al. (2017) first cast Ti_3_C_2_Tx on a flexible polyimide platform by solid-solution casting and applied Ti_3_C_2_Tx in the field of gas sensors, as shown in Figure 1A. The concentrations of ethanol, methanol, ammonia, and acetone were measured at room temperature. The efficacy for ammonia sensing was significantly higher than for the other VOCs. This is because the surface of Ti_3_C_2_Tx has abundant functional groups (Figure 1B) that react violently with ammonia gas to increase the resistance change by up to 20%, thus improving the sensing performance. Many factors affect the gas sensing performance of pristine Ti_3_C_2_Tx sensors, such as the film thickness (Kim et al., 2019), MAX phase precursor (Shuck et al., 2019), and oxidation degree (Huang Mochalin, 2020). However, despite optimization of these factors, it is difficult to efficiently and stably detect various VOC gases by relying on pure Ti_3_C_2_Tx. Therefore, compounding Ti_3_C_2_Tx with other materials and functionalizing Ti_3_C_2_Tx to improve the gas-sensing performance and selectivity of Ti_3_C_2_Tx sensors for VOC gases has also attracted increasing attention.

## Ti_3_C_2_Tx composites

To improve the sensing performance of Ti_3_C_2_Tx for VOCs gases, the combination of Ti_3_C_2_Tx with other materials has attracted much attention. Ti_3_C_2_Tx has been combined with various types of materials, such as metal oxides, graphene, and polymers, as shown in Table 2.

**TABLE 2:** Gas sensing performances of Ti3C2Tx-based gas sensors.

Ti_3_C_2_Tx composites	VOCs gas	Conc. (ppm)	Operating Temo(°C)	Response (%)	Response/Recovery time (s/s)	References
ZnSnO_3_/Ti_3_C_2_Tx	HCHO	100	RT	194.7	6.2/5.1	Sima et al. (2022)
Ti_3_C_2_T*x*/Co_3_O_4_	HCHO	10	RT	9.2	83/5	Zhang et al. (2021)
rGO/N-Ti_3_C_2_T*x*/TiO_2_	HCHO	20	RT	132	N/A	Wang et al. (2020)
Ti_3_C_2_T*x*/SnO-SnO_2_	C_3_H_6_O	100	RT	12.1	18/9	Wang et al. (2021)
Ti_3_C_2_T*x*/W_18_O_49_	C_3_H_6_O	0.17	300	1.4	5.6/6	Sun et al. (2020)
Ti_3_C_2_T*x*/rGO/CuO	C_3_H_6_O	100	RT	52.09	6.5/7.5	Liu et al. (2021a)
α-/γ-Fe_2_O_3_/ex-Ti_3_C_2_Tx	C_3_H_6_O	100	255	215.2	13/8	Huang et al. (2022)
Ti_3_C_2_T*x*/WSe_2_	C_2_H_5_OH	40	RT	24	9.7/6.6	Chen et al. (2020a)
Ti_3_C_2_T*x*/SnO_2_	C_2_H_5_OH	10	230	5	14/26	Wang et al. (2022b)
Ti_3_C_2_T*x*/Co_3_O_4_	C_2_H_5_OH	50	RT	190	50/45	Bu et al. (2022)
Ti_3_C_2_T*x*/polyaniline	C_2_H_5_OH	200	RT	41.1	0.4/0.5	Zhao et al. (2019)
Ti_3_C_2_T*x*/SnO_2_	C₆H_15_N	50	140	33.9	N/A	Liang et al. (2022)
Ti_3_C_2_T*x*/Cu_2_O	C₆H_15_N	10	RT	181.6	1,062/74	Zhou et al. (2022)
Ti_3_C_2_T*x*/In_2_O_3_	CH_3_OH	5	RT	29.6	6.5/3.5	Liu et al. (2021b)
S-Ti_3_C_2_T*x*	C_7_H_8_	10	RT	59.1	N/A	Shuvo et al. (2020)
Ti_3_C_2_T*x*/Fe_2_ (MoO_4_)_3_	C_4_H_10_	100	RT	43.1	18/24	Zou et al. (2020)

### Ti_3_C_2_Tx/metal oxide gas sensors

Metal oxides are sensitive and selective and can be used to prepare composite materials with high gas-sensing properties. The improved performance plausibly originates from the PN junction or PP junction formed by the combination of two different materials, Ti_3_C_2_Tx and a metal oxide. Many studies have been conducted on composites of Ti_3_C_2_Tx with metal oxides (Fe_2_O_3_, Co_3_O_4_, ZnSnO_3_, Cu_2_O, In_2_O_3_, and W_18_O_49_) for detecting VOCs.

Huang et al. uniformly deposited porous bi-phasic α-/γ-Fe_2_O_3_ nanoparticles on the surface and interlayer of Ti_3_C_2_Tx by solvothermal and high-temperature calcination and synthesised a stable α-/γ-Fe_2_O_3_/ex-Ti_3_C_2_Tx-X gas sensor material for acetone detection. The composite gas sensor had a good response to acetone (the response value was 215.2 for 100 ppm acetone at 255°C, and the response and recovery time were 13 and 8 s, respectively). The improved performance originates from the large number of empty cationic sites on the α-/γ-Fe_2_O_3_ surface, which can serve as strong adsorption sites for acetone. The α-/γ-Fe_2_O_3_/ex-Ti_3_C_2_Tx-X composites possess more surface defects, functional groups, porosity, and heterojunction interfaces than conventional Ti_3_C_2_Tx, which facilitates the interaction of acetone molecules with the active sites (Huang et al., 2022).

Composites of semiconductor metal oxides and Ti_3_C_2_Tx materials have also attracted much attention. (2022) successfully synthesised p-type semiconductor materials by combining Co_3_O_4_ and Ti_3_C_2_Tx, where Co_3_O_4_ was intercalated into the interlayer structure of Ti_3_C_2_Tx to form numerous hybrid heterojunctions. Intercalation significantly increased the specific surface area and gas adsorption sites of the material, thereby improving the gas-sensing performance. Zhang et al. (2021) also found that the ability of Ti_3_C_2_Tx/Co_3_O_4_ composite to respond and recover also improved with the increase of bending angle, which is of great significance for the study of flexible wearable sensors that can monitor human health in real time. Using facile electrostatic self-assembly and hydrothermal synthesis, Sima et al. (2022) successfully prepared ZnSnO_3_/Ti_3_C_2_Tx composites, which exhibited good gas-sensing properties for the detection of formaldehyde, because the ohmic contact between ZnSnO_3_ and Ti_3_C_2_Tx formed a small Teky barrier, and the work function between Ti_3_C_2_Tx and -OH (3.9 eV) was lower than that of ZnSnO_3_ (5.17 eV). According to the principle of Fermi level balance, a large number of electrons is transferred between the ZnSnO_3_ nanotubes and Ti_3_C_2_Tx to reach a relatively balanced state. More electrons will be adsorbed by oxygen on the surface of the ZnSnO_3_ nanoparticles, resulting in thickening of the electron depletion layer; thus, the resistance change will also increase, and the sensitivity of the sensor will also increase as the resistance change becomes more pronounced. Furthermore, the faster response and recovery are due to the synergistic effect between the two materials, which accelerates the separation rate of hole–electron pairs.

### Ti_3_C_2_Tx/rGO gas sensors

Graphene and Ti_3_C_2_T_x_ are both emerging two-dimensional materials with similar structures. Combining these two materials can enhance their properties through synergy. Liu et al. (2021a) fabricated a Ti_3_C_2_T_x_/rGO/CuO three-dimensional aerogel sensor material by using a one-step hydrothermal method. The material showed good acetone-sensing performance (the response value to 100 ppm acetone at room temperature was 52.09, and the response and recovery times were 6.5 and 7.5 s, respectively) and stability. The good response is mainly because the 3D porous network structure of Ti_3_C_2_T_x_/rGO/CuO prevents stacking of the composites, which exposes a larger surface area and provides more adsorption sites for O_2_ and acetone gas. As a second factor, acetone-sensing is related to the p-p junction formed at the interface owing to the different work functions of the three materials. In addition, the large number of functional groups on the surface of Ti_3_C_2_T_x_ form strong hydrogen bonds with acetone gas, the interaction force between the composite material and acetone molecules is enhanced, and the hole concentration is increased, leading to improved gas-sensing performance.

### Ti_3_C_2_Tx/polymer gas sensors

Conductive polymers are low-cost with excellent electrical conductivity and are considered potential gas sensing materials. Polyaniline (PANI) is extensively used in polymer gas sensors, where the material itself and its mixtures show excellent NH3 gas sensing performance. At present, Zhao et al. are the only ones that have prepared Ti_3_C_2_Tx/polymer composites by low-temperature *in situ* polymerisation. They found that the composites have good gas sensitivity to gaseous ethanol as a VOC (response rate to 200 ppm ethanol gas at room temperature is 41.1, with response and recovery times of 0.4 and 0.5 s, respectively). The incorporation of PANI effectively inhibited the interlayer aggregation of Ti_3_C_2_Tx, thereby exposing a larger surface area and more functional groups (–O, –OH, and–F groups), all of which increased the resistance of the composite when exposed to ethanol. Thus, the gas-sensing performance can be improved by improving the gas adsorption ability (Zhao et al., 2019).

## Functionalized Ti_3_C_2_Tx

In addition to compounding with other materials, methods of functionalizing Ti_3_C_2_Tx materials using single-atom functionalization and surface treatments are attracting increasing attention.

**FIGURE 2:**
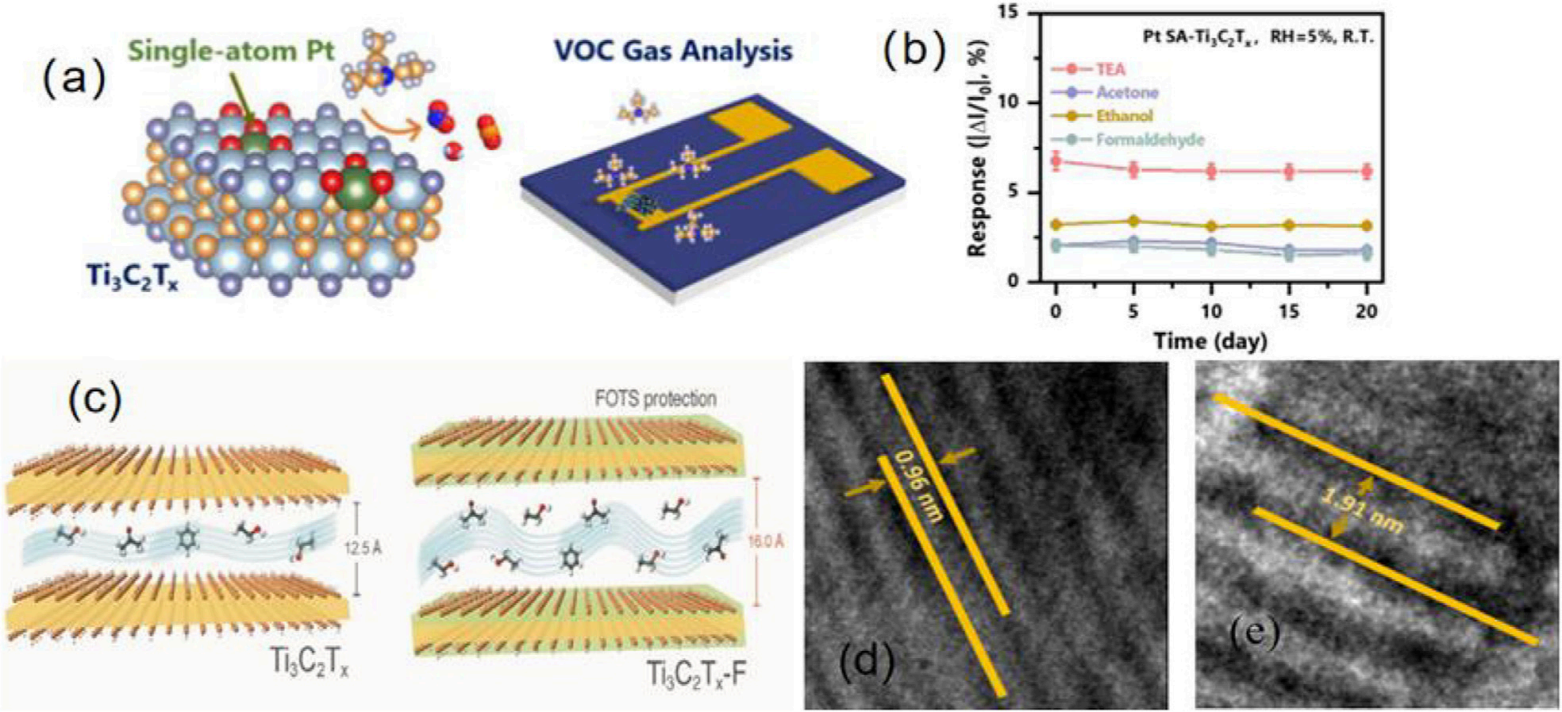
**(A)** Modified Pt atoms into sensors prepared in Ti3C2Tx. **(B)** Response rate of Pt-Ti3C2Tx sensors to various VOCs gases at room temperature for 20 consecutive days (adapted from Zong et al., 2022). **(C)** Interlayer distance between Ti3C2Tx materials before and after incorporation into FOTS. (adapted from Chen et al., 2020a) **(D–E)** Interlayer spacing of Ti3C2Tx materials before and after incorporation of S elements was observed under TEM (adapted from Shuvo et al., 2020).

As shown in Figure 2A, Zong et al. modified the surface of Ti_3_C_2_Tx with single-atom Pt (Pt SA); the resulting sensor could detect triethylamine (TEA) at levels as low as 14 ppb. The highly catalytically active and uniformly distributed Pt SA had a chemical sensitisation effect, and the excellent adsorption of Pt SA on TEA was the main reason for the improved gas-sensing performance of the sensor. Furthermore, as shown in Figure 2B, the Pt-Ti_3_C_2_Tx sensor exhibited good stability in the detection of various VOC gases at room temperature. Based on density functional theory, it was proven that metal single-atom catalyst doping can improve charge transfer in VOC gases during the adsorption process in a pioneering study on the application of metal single-atom catalysts in the field of MXene nanosheet sensors (Zong et al., 2022).

Ti_3_C_2_Tx sensors are unstable in humid environments. To solve this problem, Chen et al. (2020b) embedded fluoroalkyl silane (FOTS) on the surface of Ti_3_C_2_Tx to reduce its surface energy and achieve hydrophobic effects. Ti_3_C_2_Tx-F exhibited good hydration stability, good tolerance in acid/base solutions, and Ti_3_C_2_Tx-F detects 120 ppm ethanol gas at room temperature, showing good repeatability and fast response/recovery speed (39 s/139 s). As shown in Figure 2C, the interlayer distance of the functionalized Ti_3_C_2_Tx is larger, which can adsorb more VOCs molecules. And it is also found that the Ti-O bond length increases from 2.26 Å to 2.57 Å due to the attractive force between the oxygen and the hydrogen atoms of the ethanol, causing the adjacent oxygen atoms of the ethanol molecule to pull outward from the layer. This indicates that the gas sensing performance of Ti_3_C_2_Tx-F material will be enhanced with the adsorption of ethanol molecules. In addition, the Ti_3_C_2_Tx-F sensor can still monitor ethanol gas well in an environment with a relative humidity of 80%. This also puts forward a new idea to solve the shortcomings of Ti_3_C_2_Tx sensor, which is easy to oxidize and has poor stability in humid environment.

Shuvo et al. uniformly doped S atoms into the surface and interlayers of Ti_3_C_2_T_x_, where the responses to toluene at 1 and 50 ppm were 214% and 312%, respectively, which were 2–3 times the response of conventional Ti_3_C_2_T_x_. The TEM images in Figures 2D,E show that, after the incorporation of S atoms, the interlayer distance of the sensor material expanded significantly, thereby improving the gas sensing performance of the sensor. Furthermore, the S-Ti_3_C_2_T_x_ sensor remained stable after 30 days of continuous exposure and exhibited good repeatability over 10 consecutive cycles (Shuvo et al., 2020).

## Modification mechanism

In summary, the composite and functional methods are used to improve the gas-sensing performance of Ti_3_C_2_Tx sensor materials to VOCs gas. It is not difficult to find that although the methods are different, the modification mechanism is roughly the same. After summarizing, the author found that the modification mechanism is mainly as follows: ① Inhibiting the aggregation of Ti_3_C_2_Tx materials resulting in obtaining more surface area and more abundant functional groups; ② Improving the interaction force between the sensor material and gas molecules, and so accelerating the air The separation rate of the hole-electron pair; ③ increasing the thickness of the electron depletion layer, causing the larger channel for electron flow and thereby improving the sensitivity of the resistance change; ④ compounding with the n-type material to form a non-uniform p-n junction, making the two materials with different work functions connect together (since the Fermi level needs to be kept at the same level, electron transfer will occur between them, thereby a built-in electric field and a Schottky barrier will be formed). ⑤ Introducing other atoms to improve the charge transfer during the adsorption process. All of these reasons can effectively improve the sensing performance of the sensor, which also provides ideas for the discovery of new sensor materials in the future.

## Conclusion

The research status of gas sensors based on Ti_3_C_2_Tx in recent years was reviewed, demonstrating that the modification of Ti_3_C_2_Tx by compounding with other materials, surface modification, and single-atom doping can effectively improve the gas-sensing performance of Ti_3_C_2_Tx-based gas sensors. Combining other materials into the surface and interlayer structure of Ti_3_C_2_Tx can increase the interlayer spacing of the structure to expose a larger specific surface area, provide more active sites for target gas molecules, enhance the adsorption capacity of the sensor, and improve the sensitivity. Using density functional theory, it has been proven that metal single-atom catalyst doping can improve charge transfer in VOC gases during the adsorption process, which provides insight for developing high-performance Ti_3_C_2_Tx-based gas sensors. We hope that our work will provide guidance for the development of new Ti_3_C_2_Tx-based gas-sensor materials in the future.
